# Early Transcriptional Adaptation of *Candidozyma* (*Candida*) *auris* During Interaction with *Pseudomonas aeruginosa*

**DOI:** 10.3390/jof12100758

**Published:** 2026-10-09

**Authors:** Aliz Bozó, Ágnes Jakab, Andrea Harmath, Ágota Zsófia Ragyák, Zoltán Tóth, Andrew M. Borman, Fruzsina Kovács, Bence Balázs, Noémi Balla, László Majoros, Renátó Kovács

**Affiliations:** 1 Department of Medical Microbiology, Faculty of Medicine, University of Debrecen, 4032 Debrecen, Hungary; bozo.aliz@med.unideb.hu (A.B.); jakab.agnes@med.unideb.hu (Á.J.); harmath.andrea@med.unideb.hu (A.H.); toth.zoltan@med.unideb.hu (Z.T.); kovacs.fruzsina18@med.unideb.hu (F.K.); balazs.bence@med.unideb.hu (B.B.); balla.noemi@med.unideb.hu (N.B.); major@med.unideb.hu (L.M.); 2 Medical Microbiology, Clinical Centre, University of Debrecen, 4032 Debrecen, Hungary; 3 Doctoral School of Pharmaceutical Sciences, University of Debrecen, 4032 Debrecen, Hungary; 4 Agilent Atomic Spectroscopy Partner Laboratory, Department of Inorganic and Analytical Chemistry, University of Debrecen, 4032 Debrecen, Hungary; ragyak.agota.zsofia@science.unideb.hu; 5 UK National Mycology Reference Laboratory, UK Health Security Agency, Science Quarter, Southmead Hospital, Bristol BS10 5NB, UK; andy.borman@nbt.nhs.uk; 6 Medical Research Council Centre for Medical Mycology (MRC CMM), University of Exeter, Exeter EX4 4QD, UK

**Keywords:** *Candida auris*, *Pseudomonas aeruginosa*, polymicrobial infection, iron, metal homeostasis, transcriptomics, in vivo

## Abstract

Bacterial–fungal polymicrobial infections are clinically important, but the early response of *Candidozyma* (*Candida*) *auris* to *Pseudomonas aeruginosa* remains incompletely understood. We investigated early fungal growth, extracellular glucose and metal availability, transcriptional adaptation, and pathogen burden using in vitro co-culture, RNA sequencing, nutrient measurements, and a murine co-infection model. Within 2 h, *C. auris* increased from 3.3 ± 0.6 × 10^6^ to 7.1 ± 0.8 × 10^6^ colony-forming units (CFU)/mL in monoculture, but reached only 4.9 ± 0.4 × 10^6^ CFU/mL in co-culture. At 2 h, extracellular glucose concentrations were comparable between monoculture and co-culture despite reduced accumulation of viable *C. auris* cells in co-culture. Transcriptomics identified 210 upregulated and 124 downregulated genes, indicating remodelling of translation, carbon metabolism, stress responses, lipid metabolism, and cell-surface organization. Although iron-homeostasis genes were regulated, extracellular iron and zinc did not differ significantly at 2 h. Subsequently, extracellular iron concentration was significantly lower in co-culture at 4 h, whereas a reduction in zinc concentration at 6 h did not remain statistically significant. In vivo, co-infection reduced *C. auris* kidney burden by 1.4-log_10_, without significantly altering bacterial burden. These findings show that early bacterial interaction is associated with reduced fungal proliferation and induces transcriptional changes before detectable effects in extracellular iron and zinc, supporting a multifactorial interaction involving metabolic adaptation and subsequent environmental changes.

## 1. Introduction

Bacterial–fungal polymicrobial infections are increasingly recognized as significant contributors to healthcare-associated morbidity and mortality [1]. *Pseudomonas aeruginosa* and *Candida* species frequently coexist in device-associated infections, wounds, and respiratory diseases, including those associated with cystic fibrosis [2,3]. However, most studies of these interactions have focused on *Candida albicans*, and their findings may not be applicable to non-*albicans* species such as the emerging multidrug-resistant *Candidozyma* (*Candida*) *auris* [4,5].

In *C. albicans*, antagonistic interactions with *P. aeruginosa* involve diffusible signalling molecules, secreted metabolites, and competition for nutrients [6,7]. Recent studies have demonstrated antagonism between *C. auris* and *P. aeruginosa* and suggest that *C. auris* may respond differently than *C. albicans* to bacterial signals [4,7]. Despite these findings, the early transcriptional response of *C. auris* during direct interaction with *P. aeruginosa* remains poorly characterized.

Multiple mechanisms likely contribute to this interaction. *P. aeruginosa* produces quorum-sensing molecules, phenazines, and siderophores, with their production and activity influenced by environmental factors such as iron availability [8,9]. Iron supplementation partially restores *C. auris* growth during interaction with *P. aeruginosa*, indicating a role for extracellular iron, whereas increased glucose availability alone does not eliminate bacterial inhibition of fungal growth [4]. These findings indicate a multifactorial interaction involving bacterial signals and competition for nutrients and micronutrients. However, the relationship between these environmental changes and the early fungal transcriptional response remains unclear [4,8,9,10].

We hypothesized that the altered early proliferation of *C. auris* during interaction with *P. aeruginosa* is associated with specific metabolic and transcriptional adaptations. To investigate these responses, we employed growth analysis, measurements of extracellular glucose and metals, RNA sequencing, and an immunocompromised murine co-infection model. This integrated approach allowed us to examine the relationship between early fungal transcriptional changes and extracellular nutrient availability, as well as to assess whether the antagonistic interaction observed in vitro corresponded to altered fungal burden in vivo.

## 2. Materials and Methods

### 2.1. Isolates

The *C. auris* isolate 12 (NCPF 8973) was used in all experiments [11]. The strain was obtained from the National Mycology Reference Laboratory (Bristol, United Kingdom) and maintained on yeast extract–peptone–dextrose (YPD) agar prepared from commercially available YPD Broth (VWR Life Science, Debrecen, Hungary) supplemented with 2% (*w*/*v*) agar. According to the manufacturer’s formulation, the prepared YPD medium contains 10 g/L yeast extract, 20 g/L peptone, and 20 g/L glucose (2% *w*/*v*). For co-culture experiments, the *P. aeruginosa* PAO1 reference strain was used. The strain was maintained and cultured on Columbia agar supplemented with 5% sheep blood (Clinichem, Budapest, Hungary).

### 2.2. Co-Culture Experiments, Growth Analysis, Microscopy and Glucose Quantification

For monomicrobial growth, *C. auris* was cultured on YPD agar for 24 h at 37 °C, inoculated into 5 mL YPD broth, and incubated at 30 °C for 18 h with shaking (140 rpm). Exponentially growing cells were diluted to an OD_640_ of 0.1 in 20 mL fresh YPD and incubated at 37 °C with shaking (140 rpm). After 4 h, cultures reached an OD_640_ of 0.5 ± 0.04 (0.5 × 10^7^ ± 0.06 × 10^7^ cells/mL), at which point co-culture experiments were initiated. *P. aeruginosa* was grown on Columbia agar supplemented with 5% sheep blood for 24 h at 37 °C, resuspended in sterile physiological saline, and adjusted to an OD_640_ of 0.05 to standardize the bacterial inoculum across independent experiments. Dual-species cultures were established by adding the standardized *P. aeruginosa* suspension to exponentially growing *C. auris* cultures after 4 h of fungal growth. The viable *P. aeruginosa* concentration in the resulting co-culture was determined by CFU enumeration at the time of bacterial addition (0 h). *P. aeruginosa* monoculture controls were prepared by inoculating the same bacterial suspension into YPD alone. The time of bacterial addition was defined as 0 h. Cultures were incubated at 37 °C with shaking (140 rpm) for up to 8 h.

*C. auris* monocultures and *C. auris*–*P. aeruginosa* co-cultures were examined by propidium iodide (PI) staining at 0 and 2 h. Briefly, 999 µL of culture was supplemented with 1 µL of 20 mM PI (Thermo Fisher Scientific, Waltham, MA, USA) and incubated at 37 °C for 30 min. Subsequently, 10 µL was mounted on a microscope slide and examined using a Zeiss Axioskop 2 mot microscope coupled with a Zeiss Axiocam 212 camera (Zeiss, Jena, Germany) using phase-contrast and fluorescence microscopy [12]. Image acquisition was performed using Zeiss ZEN lite 3.13 software. Heat-inactivated *C. auris* cells served as a positive control. Experiments were performed in three independent biological replicates.

Viable cell counts of each microorganism in mono- and dual-species cultures were determined by serial dilution and species-selective plating. *C. auris* CFUs were enumerated on Sabouraud dextrose agar (SDA) supplemented with 50 mg/L chloramphenicol to suppress bacterial growth, whereas *P. aeruginosa* CFUs were enumerated on Mueller–Hinton agar supplemented with 8 mg/L amphotericin B to suppress fungal growth. Plates were incubated at 37 °C for 48 h before colony enumeration.

Extracellular glucose concentrations were measured during the 2 h incubation using a glucose oxidase assay [13,14]. The reaction mixture contained 0.76 mmol/L 4-aminoantipyrine, 11 mmol/L phenol, 4 kU/L glucose oxidase, and 1 kU/L horseradish peroxidase in 0.1 mol/L sodium–potassium phosphate buffer (pH 6.6). Absorbance was recorded at 500 nm immediately after addition of 10% (*v*/*v*) sample. Cell-free culture supernatants were collected at the end of the 4-h *C. auris* pre-growth period, immediately before *P. aeruginosa* addition (defined as 0 h), and 2 h after bacterial addition. Glucose concentrations were analysed directly without normalization to CFU counts and are reported as g/L. The nominal glucose concentration of the fresh YPD medium was 20 g/L.

### 2.3. RNA-Seq and Transcriptome Analysis

Following 4 h of pre-growth of *C. auris* in fresh YPD medium, *P. aeruginosa* was added to initiate co-culture. Samples from *C. auris* monocultures and co-cultures were collected 2 h later, corresponding to 6 h after the initiation of fungal cultivation, and washed with sterile physiological saline by centrifugation at 3000× *g* for 10 min at 4 °C. The resulting cell pellets were used for RNA extraction and RNA-seq analysis. The 2-h time point was chosen to capture the early transcriptional response of *C. auris* to *P. aeruginosa*. At this stage, a significant difference in viable fungal CFU accumulation was already detectable between monoculture and co-culture, while no significant co-culture-associated differences in bulk extracellular glucose, iron, or zinc concentrations had yet emerged.

Total RNA was extracted from lyophilized cells derived from three biological replicates per condition. Extractions were performed using TRI Reagent (Merck, Budapest, Hungary) according to the method of Chomczynski (1993) [15]. RNA quality and quantity were assessed using a Bioanalyzer (Agilent Technologies, Santa Clara, CA, USA). All samples had RNA integrity number (RIN) values between 8.2 and 9.0 and RNA concentrations of at least 1.7 µg/µL. RNA library preparation and sequencing were performed by the Genomic Medicine and Bioinformatics Core Facility (Department of Biochemistry and Molecular Biology, Faculty of Medicine, University of Debrecen, Debrecen, Hungary). As the first step of library preparation, polyadenylated RNA was enriched using oligo(dT)-conjugated magnetic beads. This enrichment selectively retained eukaryotic poly(A)+ mRNA while substantially depleting non-polyadenylated RNA species, including fungal ribosomal RNA and the majority of bacterial RNA present in the mixed-species samples. Libraries were subsequently sequenced on an Illumina NextSeq 2000 platform (Illumina, San Diego, CA, USA) using single-end 100-bp sequencing. Approximately 17.7 to 29.3 million sequencing reads were generated per sample. Read quality was assessed using the FastQC (version 0.12.1) software package (http://www.bioinformatics.babraham.ac.uk/projects/fastqc; accessed on 1 December 2024). Sequencing reads were aligned to the *C. auris* B8441 reference genome (genome version GCA_002759435.2) using HISAT2 v2.1 and BAM files were generated. Across the six libraries, 92.9–93.1% of reads aligned to the *C. auris* B8441 reference genome in monoculture samples and 92.2–93.2% in co-culture samples. Downstream analysis was performed using StrandNGS 4.0 software. Gene-level read counts were normalized using the DESeq normalization method implemented in StrandNGS 4.0. Principal component analysis of the samples was performed based on normalized expression values using StrandNGS 4.0.

Differentially expressed genes were identified using a moderated *t*-test with Benjamini–Hochberg false discovery rate (FDR) correction for multiple testing. Genes with an FDR-adjusted *p*-value < 0.05 and a fold-change magnitude of at least 1.5 were considered differentially expressed. Following the signed fold-change convention used by Strand NGS, genes with FC > 1.5 were classified as upregulated and those with FC < −1.5 as downregulated.

Functional enrichment analysis was performed using the Candida Genome Database Gene Ontology Term Finder (http://www.candidagenome.org/cgi-bin/GO/goTermFinder; accessed on 1 December 2024) and FungiDB (https://fungidb.org/fungidb/app/; accessed on 1 December 2024) to identify enriched biological processes, cellular components, molecular functions, and KEGG (Kyoto Encyclopedia of Genes and Genomes) metabolic pathways. Figures were generated using the SRPlot platform (http://www.bioinformatics.com.cn/SRplot; accessed on 1 December 2024).

### 2.4. Validation of RNA-Seq Results by RT-qPCR

RT-qPCR analysis was performed as previously described [7,12,14]. Following DNase I treatment (Merck, Budapest, Hungary), the Luna Universal One-Step RT-qPCR Kit (New England Biolabs, Ipswich, MA, USA) was used according to the manufacturer’s protocol, with oligonucleotide primer pairs listed in Table S1. All experiments were performed in technical triplicates and repeated in three independent biological experiments for each sample. Relative transcription levels were quantified using the ΔΔCP method, with *ACT1* (B9J08_000486) used as the reference gene. *ACT1* has previously been identified among the most stable candidate reference genes in *C. auris* and has been used for normalization in independent *C. auris* RT-qPCR studies [16,17,18]. PCR amplification efficiencies were determined from raw fluorescence data using LinRegPCR according to Untergasser et al. (2021) [19]. Mean amplification efficiencies ranged from 89.37% to 95.10%, with primer-specific efficiency values provided in Table 1. Melting-curve analysis was performed to assess amplification specificity, and a single peak was observed for each primer pair.

### 2.5. Comparison with the Previously Published HSL-Responsive Transcriptomic Dataset

To compare the transcriptional response of *C. auris* to *P. aeruginosa* with that exerted by exogenous HSL, the differentially expressed genes identified in the present co-culture experiment were compared with HSL-responsive genes derived from our previous transcriptomic study [7]. In that study, *C. auris* was exposed to 100 or 200 µM HSL. It is noteworthy that no new HSL-exposure experiments or transcriptomic sequencing were performed for the present study; the previously published genes were used exclusively for comparative analysis.

### 2.6. Elemental Analysis of Cultures Using Inductively Coupled Plasma Optical Emission Spectrometry (ICP-OES)

Aliquots of *C. auris* monoculture, *P. aeruginosa* monoculture, and *C. auris*–*P. aeruginosa* co-culture were collected at 0, 2, 4, 6, and 8 h after bacterial addition and centrifuged for 5 min at 4000× *g* and 4 °C. The resulting cell-free culture supernatants were collected for determination of extracellular elemental concentrations. Supernatant samples (0.65 ± 0.04 g) were weighed into glass beakers using an analytical balance (Precisa 240A) and subjected to open-vessel wet digestion with 3 mL 65% (m/m) nitric acid and 1 mL 30% (m/m) hydrogen peroxide (reagent grade, Merck, Budapest, Hungary). Digested solutions were quantitatively transferred to volume-calibrated plastic centrifuge tubes and diluted to 10 mL with 0.1 M nitric acid prepared in ultrapure water (Synergy UV, Merck, Budapest, Hungary). Samples were stored at ambient temperature until analysis [7,12,13].

Elemental composition was determined by ICP-OES (5110 Vertical Dual View, Agilent Technologies, Santa Clara, CA, USA) equipped with an autosampler (Agilent SPS4), OneNeb nebulizer, and double-pass spray chamber. Operating conditions and analytical parameters are summarized in Table S2.

Calibration standards for Ca, K, Mg, and Na were prepared from single-element stock solutions (1000 mg/L, Scharlab, Debrecen, Hungary), while Ag, Al, B, Ba, Cd, Co, Cu, Cr, Fe, Li, Mn, Ni, Pb, Sr, and Zn standards were prepared from a multi-element stock solution (1000 mg/L, ICP IV, Merck, Budapest, Hungary). Five-point calibration curves were generated using standards diluted in 0.1 M nitric acid prepared with ultrapure water.

### 2.7. In Vivo Immunocompromised Mouse Model

Immunocompromised female BALB/c mice (22–25 g) were used for in vivo experiments. Animals were maintained in accordance with the Guidelines for the Care and Use of Laboratory Animals, and all procedures were approved by the Animal Care Committee of the University of Debrecen (permission no. 08/2024 DEMÁB).

Immunosuppression was induced by intraperitoneal cyclophosphamide (Endoxan, Baxter, Deerfield, FL, USA) administered at 150 mg/kg body weight 4 days prior to infection and 100 mg/kg 1 day before infection. Additional doses of 100 mg/kg were given on days 2 and 4 postinfection. On day 0, mice were inoculated intravenously via the lateral tail vein [20,21,22]. In monoinfection experiments, animals received either *C. auris* (1 × 10^7^ CFU in 200 μL of sterile physiological saline) or *P. aeruginosa* (1 × 10^6^ CFU in 200 μL of sterile physiological saline). Inoculum concentrations were confirmed by serial dilution and species-selective plating. *C. auris* was enumerated on Sabouraud dextrose agar supplemented with 50 mg/L chloramphenicol, whereas *P. aeruginosa* was enumerated on Mueller–Hinton agar supplemented with 8 mg/L amphotericin B.

For mixed infections, mice were co-inoculated with both organisms at the same respective doses (1 × 10^7^ CFU *C. auris* and 1 × 10^6^ CFU *P. aeruginosa*) in a total volume of 200 μL. The concentrations of both microorganisms in the mixed inoculum were independently confirmed before infection by serial dilution and species-selective plating using the same media described above. Mice were randomly assigned to three groups (n = 10 per group): (i) *C. auris* monoinfection, (ii) *P. aeruginosa* monoinfection, (iii) mixed infection. On day 6 postinfection, mice were euthanized by manual cervical dislocation. Subsequently, the kidneys were aseptically removed, weighed, and homogenized in sterile physiological saline. Serial dilutions of kidney homogenates were subjected to species-selective plating. *C. auris* was recovered and quantified on Sabouraud dextrose agar supplemented with 50 mg/L chloramphenicol to suppress bacterial growth, whereas *P. aeruginosa* was recovered and quantified on Mueller–Hinton agar supplemented with 8 mg/L amphotericin B to suppress fungal growth. Plates were incubated at 37 °C for 48 h, and microbial burdens were expressed as CFU per gram of kidney tissue. Kidneys were selected for quantitative microbial burden analysis because renal fungal burden serves as a well-established endpoint in murine models of systemic candidiasis following intravenous infection. Blood samples were not analyzed, as the study prioritized assessment of established tissue burden at day 6 post-infection rather than transient microorganisms present in the bloodstream [23,24].

### 2.8. Statistical Analysis

Growth experiments, extracellular glucose measurements, and extracellular metal determinations were performed in three independent biological experiments, and data are presented as mean ± standard deviation (SD). For growth experiments, *C. auris* CFU counts in monoculture and co-culture were compared at the 2-h interaction time point using a two-sided Welch’s *t*-test. For extracellular glucose, the primary comparison was between *C. auris* monoculture and *C. auris*–*P. aeruginosa* co-culture at the 2-h transcriptomic sampling point, with within-condition changes from 0 to 2 h analysed separately. Extracellular metal concentrations were measured in three independent biological cultures per condition, with samples collected at 0, 2, 4, 6, and 8 h. At each time point, pairwise comparisons among *C. auris* monoculture, *P. aeruginosa* monoculture, and co-culture were conducted using two-sided Welch’s *t*-tests. For each element, *p*-values were adjusted using the Holm method across all 15 pairwise comparisons, representing three comparisons at each of five time points. An adjusted *p*-value less than 0.05 was considered statistically significant. For the in vivo experiments, fungal and bacterial kidney burdens were analysed separately. *C. auris* burdens in monoinfected and co-infected animals were compared using a two-tailed Mann–Whitney U test. *P. aeruginosa* burdens in monoinfected and co-infected animals were analysed independently using the same test. Exact *p*-values were calculated, and *p* < 0.05 was considered statistically significant. All statistical analyses were performed using GraphPad Prism v10.6.1.

## 3. Results

### 3.1. Candida Auris Shows Reduced Early Proliferation During Co-Culture with Pseudomonas aeruginosa Without a Detectable Co-Culture-Specific Reduction in Extracellular Glucose

**Figure 1:**
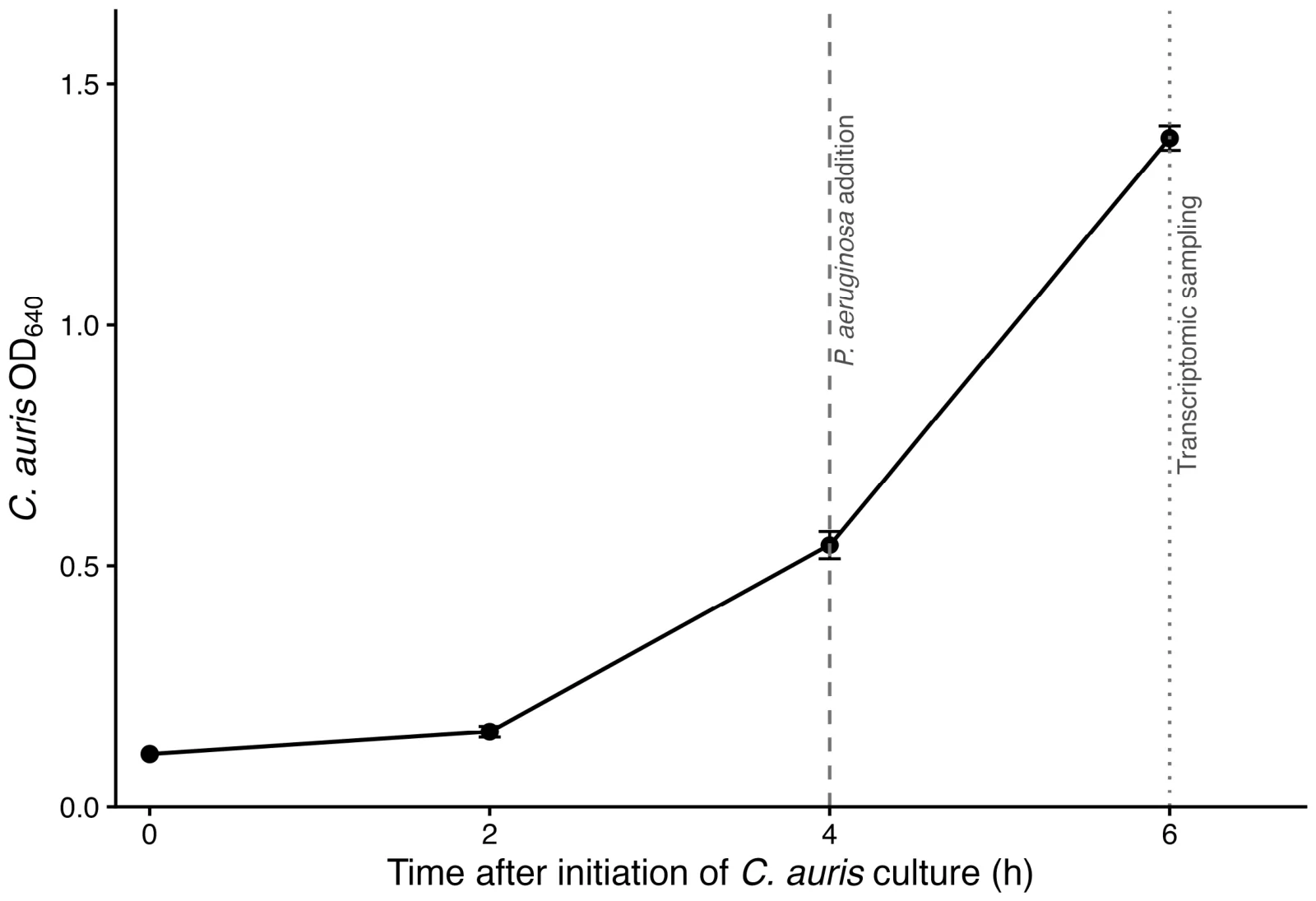
Growth curve of *C. auris* under monoculture conditions. Optical density (OD_640_) was measured at 0, 2, 4, and 6 h after the initiation of fungal culturing. The dashed vertical line at 4 h indicates the time point corresponding to *P. aeruginosa* addition in the parallel co-culture experiment, whereas the dotted vertical line at 6 h indicates the time point corresponding to transcriptomic sampling. Data are presented as means ± standard deviations.

To establish the growth status of *C. auris* at the time of bacterial addition and transcriptomic sampling, an OD_640_-based growth profile was evaluated in monoculture (Figure 1). OD_640_ increased from 0.11 at 0 h to 0.16 ± 0.01 at 2 h, 0.54 ± 0.03 at 4 h, and 1.39 ± 0.03 at 6 h after initiation of the fungal culture. As *P. aeruginosa* was added after 4 h of fungal pre-growth, the 2-h interaction period corresponded to 6 h of fungal cultivation, when the increase in OD_640_ indicated measurable fungal proliferation rather than persistence in the initial lag period.

**Figure 2:**
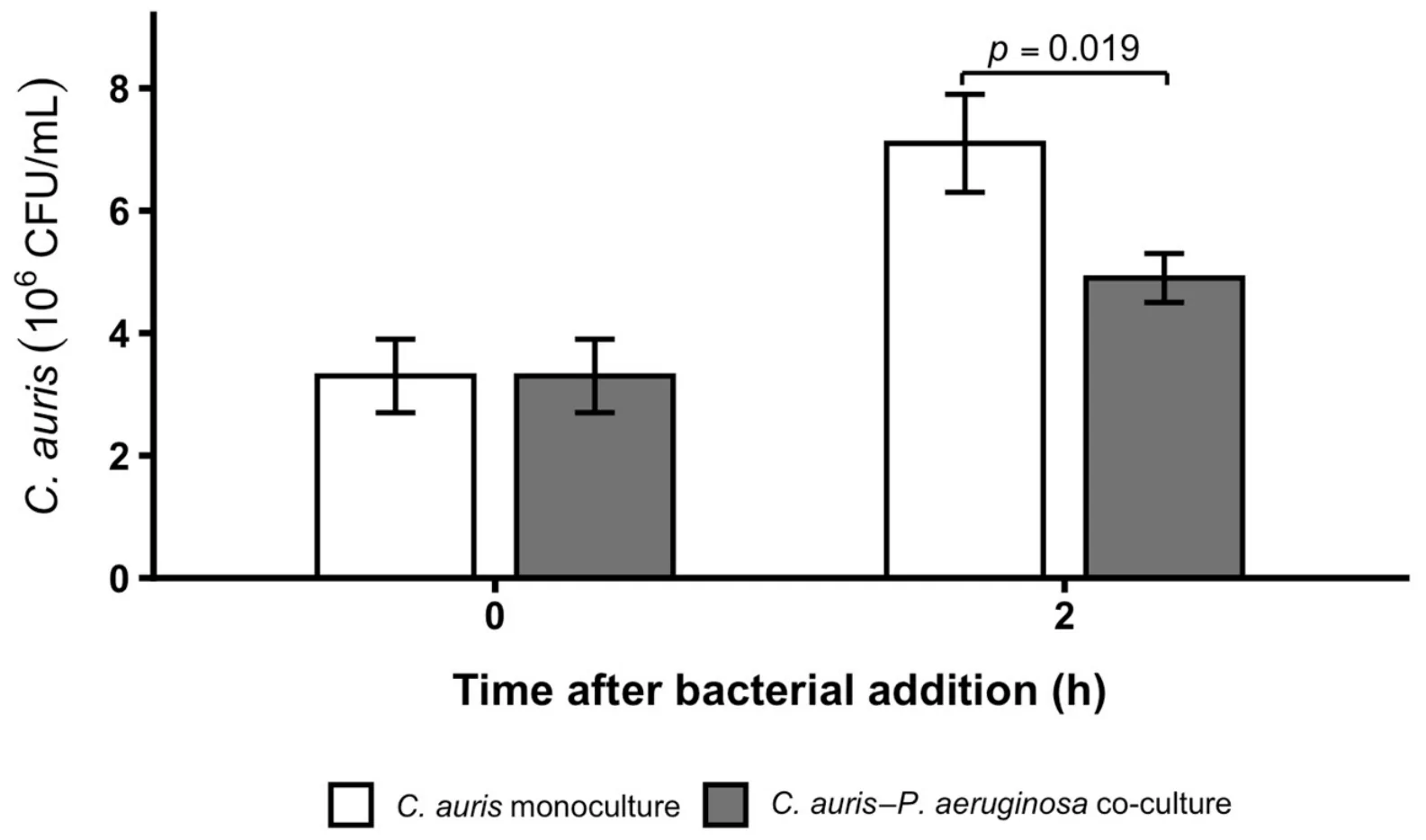
Effect of *P. aeruginosa* co-culture on the early viable cell count of *C. auris*. *C. auris* cell counts were determined at the time of *P. aeruginosa* addition (0 h), following the 4-h fungal pre-growth period, and after 2 h of incubation in monoculture or co-culture with *P. aeruginosa*. At 0 h, the *C. auris* population was 3.3 ± 0.6 × 10^6^ CFU/mL. After 2 h, the fungal population increased to 7.1 ± 0.8 × 10^6^ CFU/mL in monoculture and to 4.9 ± 0.4 × 10^6^ CFU/mL in co-culture. The *C. auris* CFU count was significantly lower in co-culture than in monoculture at 2 h (*p* = 0.019). Data are presented as mean ± SD.

Co-culture with *P. aeruginosa* was associated with reduced early *C. auris* proliferation. At the time of bacterial addition (0 h), following the 4-h fungal pre-growth period, the *C. auris* population was 3.3 ± 0.6 × 10^6^ CFU/mL. During the subsequent 2 h, the fungal population increased to 7.1 ± 0.8 × 10^6^ CFU/mL in monoculture, whereas it reached 4.9 ± 0.4 × 10^6^ CFU/mL in co-culture. At 2 h, the *C. auris* CFU count was significantly lower in co-culture than in monoculture (*p* = 0.019), corresponding to an approximately 31% lower fungal burden (Figure 2). Concurrently, *P. aeruginosa* proliferated rapidly in co-culture, increasing from 0.15 ± 0.06 × 10^7^ CFU/mL at 0 h to 2.97 ± 0.12 × 10^7^ CFU/mL at 2 h.

To determine whether the reduced *C. auris* CFU count in co-culture was associated with increased fungal cell death, PI staining was performed at 0 and 2 h. PI-positive cells were readily detected in the heat-inactivated control; however, no remarkable increase was observed in co-culture compared to monoculture at 2 h (Figure S1). Consistent results were obtained across three independent biological experiments, suggesting no substantial co-culture-associated loss of fungal viability at the transcriptomic sampling point.

Extracellular glucose concentrations were also examined at the 2-h transcriptomic sampling point. Following the 4-h *C. auris* pre-growth period, the glucose concentration was 17.22 ± 0.10 g/L at 0 h. After 2 h, glucose concentrations decreased to 15.78 ± 0.12 g/L in the *C. auris* monoculture and 15.71 ± 0.10 g/L in the co-culture with *P. aeruginosa*, with no significant difference between the two conditions (*p* = 0.589). Thus, the lower viable *C. auris* cell count observed during co-culture was not accompanied by a detectable co-culture-associated reduction in bulk extracellular glucose concentration at this time point.

### 3.2. Pseudomonas aeruginosa Induces Extensive Early Transcriptional Remodelling in Candida auris

**Figure 3:**
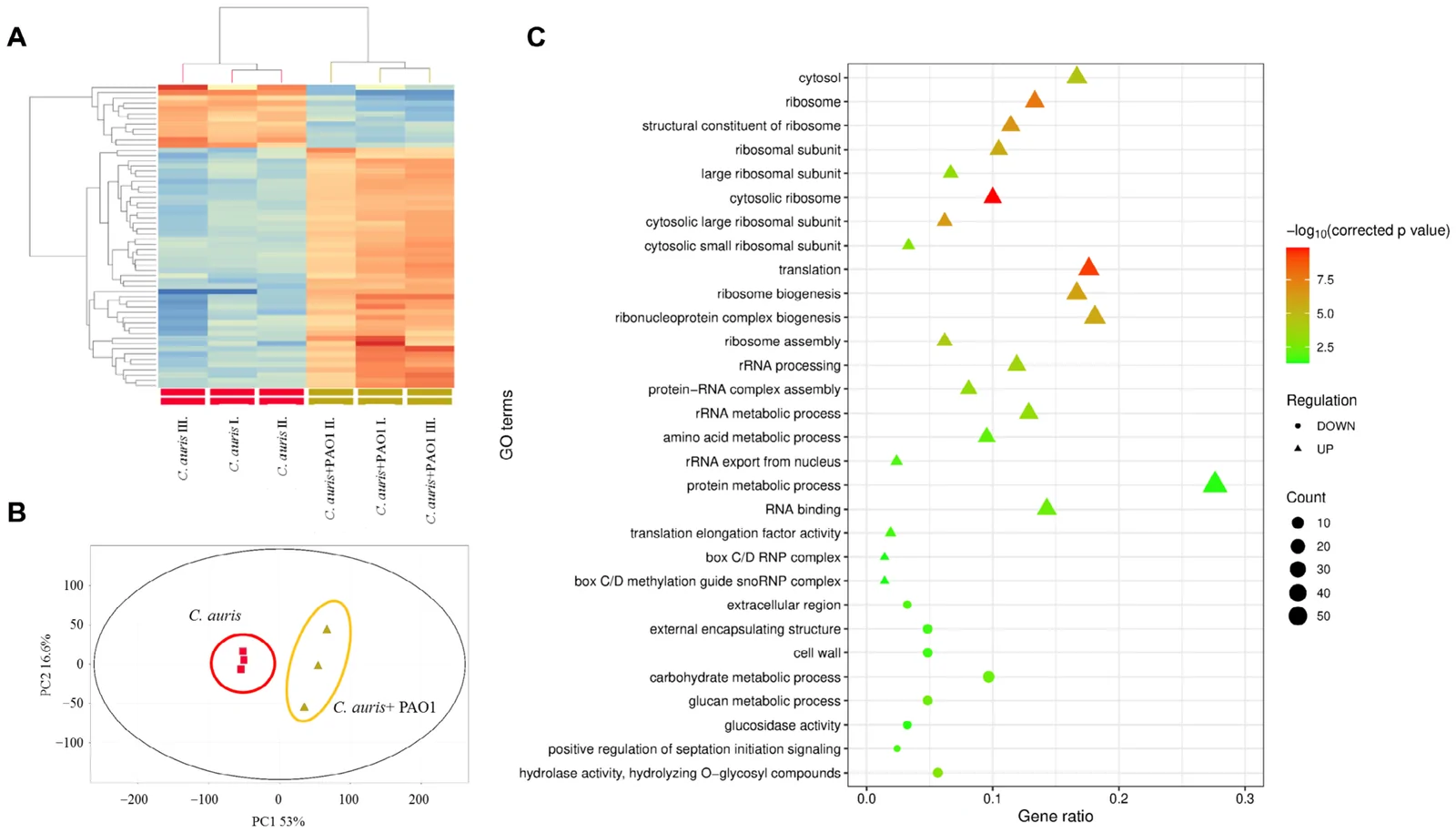
Transcriptome profiles of *C. auris* in the presence and absence of *P. aeruginosa***.** (**A**) Heat map showing transcription levels of clustered differentially expressed genes under mono- and co-culture conditions. Red and blue indicate relatively higher and lower transcription levels, respectively. (**B**) Principal component analysis (PCA) plot of three biological replicates per condition. (**C**) Gene Ontology (GO) enrichment analysis of differentially expressed genes. Gene ratio represents the number of differentially expressed genes assigned to a given GO term divided by the total number of differentially expressed genes in the corresponding regulation group (210 upregulated and 124 downregulated genes). For both upregulated and downregulated GO terms, symbol size represents the number of differentially expressed genes assigned to each GO term (Count), whereas symbol colour indicates −log_10_ of the corrected *p*-value. Triangles and circles indicate GO terms enriched among upregulated and downregulated genes, respectively. The full list of enriched GO terms is provided in Table S3.

To characterize the fungal response associated with decreased proliferation during co-culture, transcriptional changes in *C. auris* were examined 2 h after the addition of *P. aeruginosa*. Hierarchical clustering and principal-component analysis (PCA) distinctly separated *C. auris* monoculture and co-culture samples by experimental condition, with the three biological replicates clustering closely within each group (Figure 3A,B). In total, 210 genes were upregulated and 124 genes were downregulated in *C. auris* during co-culture with *P. aeruginosa* according to the differential-expression criteria defined in the Materials and Methods.

Gene Ontology (GO) enrichment analysis revealed distinct functional patterns among the upregulated and downregulated genes (Figure 3C; Table S3). Upregulated genes were strongly enriched for ribosome-associated functions, including ribosome (gene ratio [GR] = 0.133; *p* = 1.73 × 10^−8^), cytosolic ribosome (GR = 0.100; *p* = 1.30 × 10^−10^), translation (GR = 0.176; *p* = 5.17 × 10^−10^), and ribosome biogenesis (GR = 0.167; *p* = 6.63 × 10^−7^). Enrichment of rRNA-processing and ribonucleoprotein-complex-biogenesis terms further supported coordinated regulation of ribosome- and translation-associated processes. In contrast, downregulated genes were enriched for functions associated with cell-surface organization and carbohydrate metabolism, including glucan metabolic processes and hydrolase activity, hydrolyzing O-glycosyl compounds (Figure 3C; Table S3). Collectively, these results indicate that the early fungal response to *P. aeruginosa* involves extensive transcriptional remodelling of translation-associated, metabolic, and cell-surface-associated processes.

**Figure 4:**
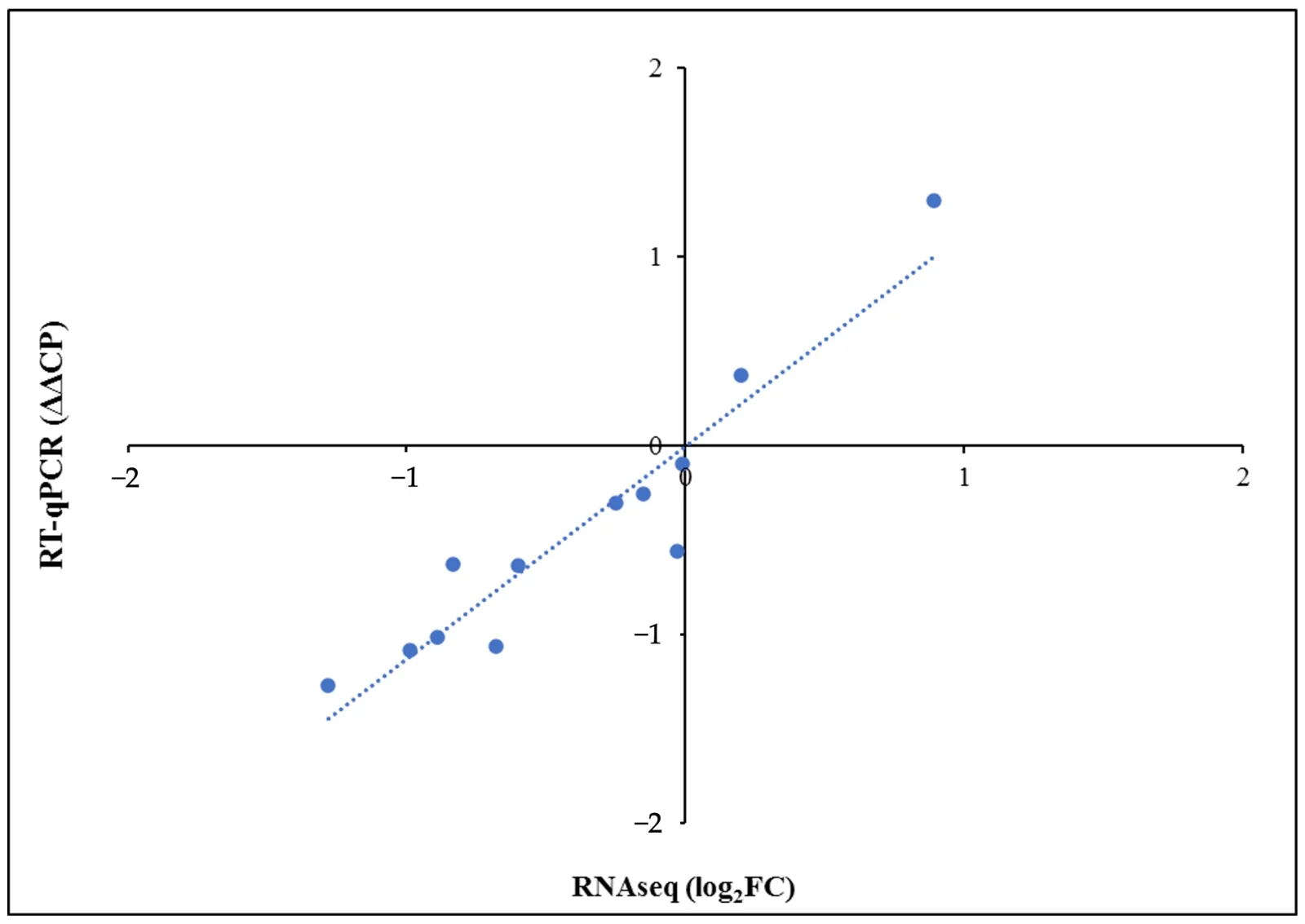
Correlation between RNA-seq and RT-qPCR expression changes in selected *C. auris* genes. Expression changes for the 12 selected genes in *C. auris* during co-culture with *P. aeruginosa* relative to *C. auris* monoculture were compared between RNA-seq and RT-qPCR analyses. RNA-seq data are expressed as log_2_ fold change (log_2_FC), and RT-qPCR data as ΔΔCP. The dotted line represents the linear regression. Pearson’s correlation coefficient was r = 0.945.

For the 12 genes selected for independent RT-qPCR validation, expression changes measured by RT-qPCR showed a strong correlation with the obtained RNA-seq results (Pearson correlation coefficient, r = 0.945; Table 1, Figure 4).

**Table 1:** RT-qPCR validation of RNA-seq results for selected *C. auris* genes. Expression changes in *C. auris* during co-culture with *P. aeruginosa* relative to *C. auris* monoculture are shown for 12 selected genes. RT-qPCR data are presented as mean ΔΔCP ± SD from three independent biological experiments. RNA-seq values are presented as log_2_ fold changes (log_2_FC). The Pearson correlation coefficient between RT-qPCR ΔΔCP and RNA-seq log_2_FC values was r = 0.945. Amplification efficiencies were calculated from raw fluorescence data using LinRegPCR and are presented as mean ± SD. Melting-curve analysis yielded a single peak for each primer pair.

*C. auris* Gene ID	*C. albicans* Gene Name	RT-qPCR ΔΔCP, Mean	SD	Amplification Efficiency, Mean ± SD (%)	RNA-Seq log_2_FC
B9J08_000486	*ACT1*	−0.093	0.067	90.37 ± 2.28	−0.013
B9J08_003245	*TUB2*	0.370	0.175	92.88 ± 5.95	0.198
B9J08_002682	*MKC1*	−0.557	0.620	89.91 ± 5.78	−0.028
B9J08_005368	*YDJ1*	1.293	0.313	89.95 ± 3.58	0.891
B9J08_003981	*MDR1*	−1.013	0.146	89.37 ± 3.02	−0.890
B9J08_003446	*RAS1*	−0.303	0.179	90.70 ± 3.66	−0.248
B9J08_000270	*UPC2*	−0.257	0.170	95.10 ± 3.82	−0.153
B9J08_003026	*ERG24*	−1.063	0.114	93.30 ± 5.16	−0.678
B9J08_001292	*SOD4*	−1.080	0.190	89.69 ± 3.55	−0.989
B9J08_000367	*ERG25*	−0.633	0.083	92.00 ± 3.58	−0.598
B9J08_003627	*HSP21*	−0.630	0.200	91.88 ± 4.18	−0.833
B9J08_004152	*HMX1*	−1.267	0.270	92.44 ± 2.44	−1.280

### 3.3. Carbon Metabolism Is Transcriptionally Remodelled During Early Co-Culture

Given the prominent transcriptional changes involving carbon-metabolism-associated pathways, genes involved in carbon metabolism were examined in greater detail. Key glycolytic regulators, including *PFK1*, *PFK2*, and *TYE7*, exhibited increased expression, while *PCK1* and *PDC11* were also differentially regulated. These findings indicate substantial changes in carbon-metabolic pathways. Furthermore, all four examined genes involved in N-acetylglucosamine (GlcNAc) utilization were significantly upregulated, including the GlcNAc uptake transporter *NGT1*, hexokinase *HXK1*, GlcNAc deacetylase *DAC1*, and GlcNAc deaminase *NAG1*. Alongside the enrichment of carbohydrate-metabolism-associated functions among downregulated genes, these results indicate extensive transcriptional reorganization of carbon metabolism during early interaction with *P. aeruginosa*. These transcriptional changes occurred despite comparable bulk extracellular glucose concentrations in *C. auris* monoculture and co-culture at the 2-h sampling point. Thus, the observed carbon-metabolic transcriptional remodelling cannot be attributed to a detectable co-culture-specific reduction in bulk extracellular glucose concentration, although alterations in glucose utilization or carbon-metabolic regulation at the cellular level cannot be excluded.

### 3.4. Early Co-Culture Induces Selective Stress-, Lipid-, Cell-Surface-, and Iron-Homeostasis-Associated Transcriptional Responses

Stress-associated genes exhibited a heterogeneous transcriptional response during co-culture. *SOD4*, *HSP21*, and the transcription factor *SFP1* were downregulated, whereas several heat-shock proteins and associated regulators, including *HSP60*, *HSP10*, *HSP90*, *SSA2*, *AHA1*, *SSB1*, *YDJ1*, *PST3*, and *HAP41*, were upregulated. Thus, co-culture was associated with selective rather than uniform regulation of stress-responsive genes.

Genes involved in lipid anabolic pathways were predominantly downregulated. This group included the ergosterol-biosynthesis-associated genes *ERG2*, *ERG24*, and *ERG25*, as well as the fatty-acid-associated genes *FAS1*, *FAD2*, and B9J08_004437. Genes associated with the biosynthesis of structural cell-wall components, such as the mannan-glucan complex, were also downregulated. In line with these changes, the adhesion-associated genes *PGA4*, *PGA7*, and *IRS4* were downregulated as well (Figure 3C; Table S3).

Iron-homeostasis-associated genes, such as *FRE10* and *HMX1*, were downregulated at the 2-h transcriptomic sampling point. At this time point, no statistically significant difference in bulk extracellular iron concentration was detected between *C. auris* monoculture and co-culture. Thus, regulation of the iron-homeostasis-associated genes *FRE10* and *HMX1* was already evident before the later numerical divergence in extracellular iron profiles.

### 3.5. Extracellular Iron and Zinc Concentrations Change Dynamically During Prolonged Co-Culture

**Figure 5:**
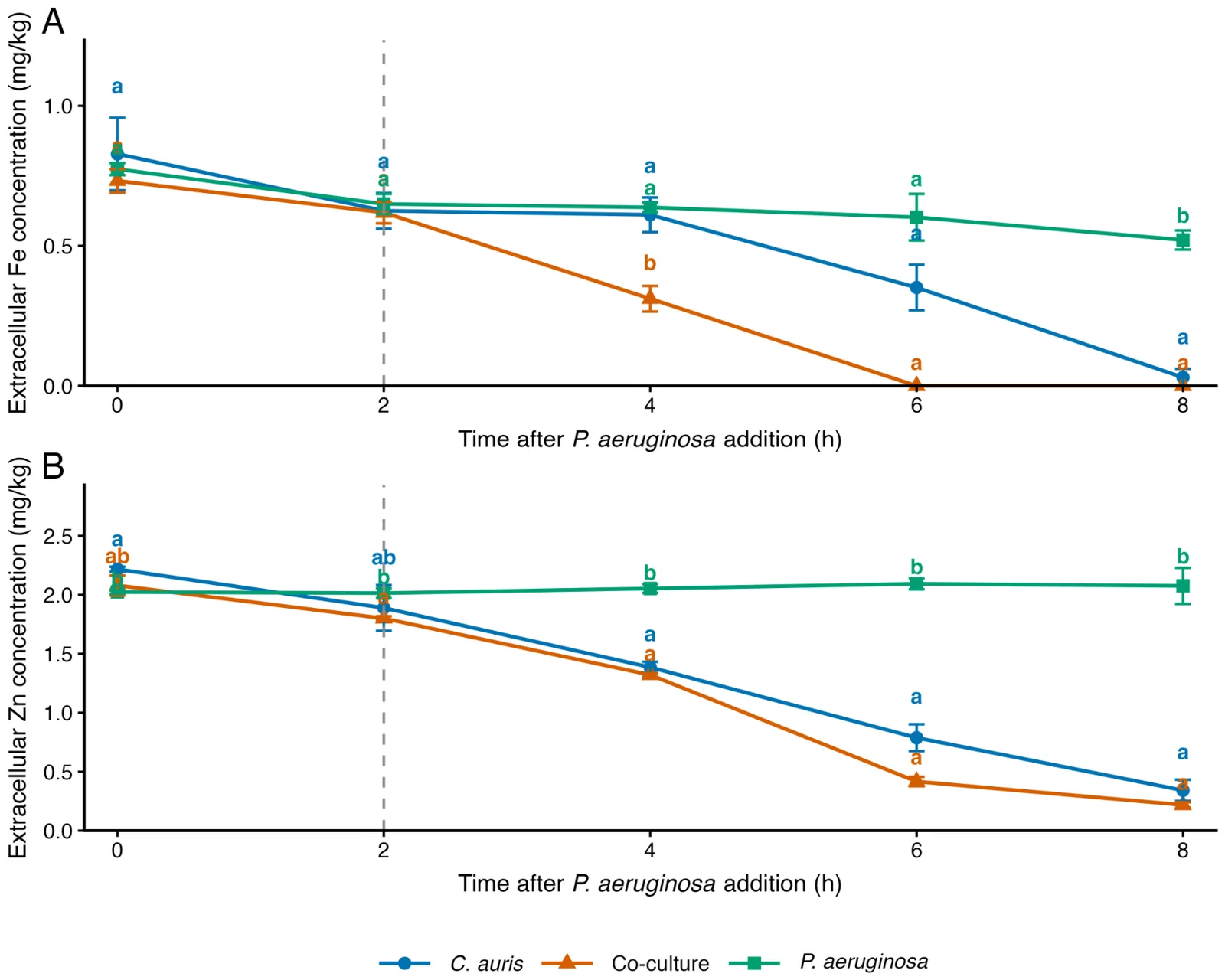
Temporal changes in extracellular iron and zinc concentrations during *C. auris*–*P. aeruginosa* co-culture. Extracellular iron (**A**) and zinc (**B**) concentrations were measured in *C. auris* monoculture, *P. aeruginosa* monoculture, and *C. auris*–*P. aeruginosa* co-culture at 0, 2, 4, 6, and 8 h after the addition of *P. aeruginosa*. Data are presented as mean ± SD from three independent biological experiments. The vertical dashed line indicates the 2-h time point used for transcriptomic sampling. At each time point, pairwise comparisons among the three culture conditions were performed using two-sided Welch’s *t*-tests, with Holm correction for multiple comparisons applied separately for each element. Lowercase letters (a, b, or ab) indicate the statistical grouping of the culture conditions at each time point: groups labelled with different letters (a vs. b) are significantly different (Holm-adjusted *p* < 0.05), whereas groups sharing the same letter or at least one letter (a vs. a, b vs. b, or a vs. ab) are not significantly different.

A comprehensive panel of elements was quantified using ICP-OES over the 0–8 h interaction period. Fe and Zn were selected for detailed analysis due to their pronounced temporal changes during co-culture compared to monoculture (Figure 5A,B). At the 2-h transcriptomic sampling point, neither extracellular Fe (0.63 ± 0.06 vs. 0.62 ± 0.04 mg/kg; Holm-adjusted *p* = 1.000) nor Zn (1.89 ± 0.19 vs. 1.80 ± 0.02 mg/kg; Holm-adjusted *p* = 1.000) differed significantly between *C. auris* monoculture and co-culture. At 4 h after *P. aeruginosa* addition, extracellular Fe was approximately 49% lower in co-culture than in monoculture (0.31 ± 0.05 vs. 0.61 ± 0.06 mg/kg; Holm-adjusted *p* = 0.040), whereas at 6 h, extracellular Zn was approximately 47% lower (0.42 ± 0.04 vs. 0.79 ± 0.11 mg/kg), although this difference was not significant after multiple-comparison correction (Holm-adjusted *p* = 0.145). These results indicate that the early transcriptional response at 2 h occurred prior to detectable co-culture-associated changes in bulk extracellular Fe and Zn concentrations.

### 3.6. The Transcriptional Response to Pseudomonas aeruginosa Differs from That Induced by Exogenous N-(3-Oxododecanoyl)-L-homoserine Lactone

**Figure 6:**
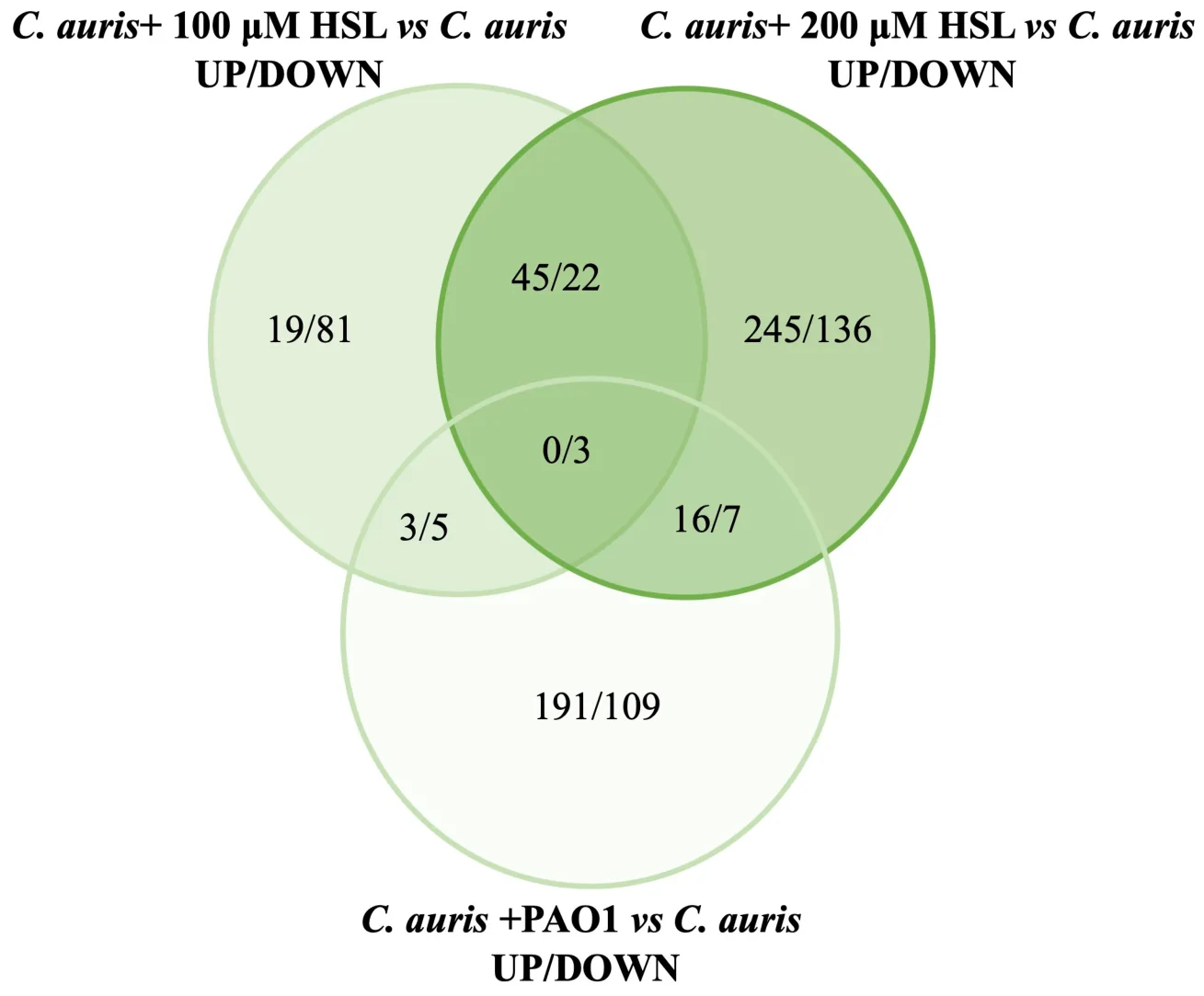
Comparison of differentially expressed genes in *C. auris* following exposure to N-(3-oxododecanoyl)-L-homoserine lactone (HSL) or co-culture with *P. aeruginosa*. The Venn diagram illustrates the overlap between upregulated and downregulated genes identified following exposure to 100 µM or 200 µM HSL and those identified during co-culture with *P. aeruginosa*. HSL-responsive gene sets were obtained from the previously published transcriptomic dataset of Kovács et al. (2025) [7].

To determine whether the transcriptional response to *P. aeruginosa* resembled that induced by HSL, the 334 DEGs identified in the present co-culture experiment were compared with HSL-responsive gene sets from our previously published transcriptomic dataset, in which *C. auris* was exposed to 100 or 200 µM HSL [7]. Only a small subset of co-culture DEGs overlapped with the HSL-responsive gene sets (Figure 6), showing limited overlap between the transcriptional responses observed under the respective experimental conditions.

### 3.7. Pseudomonas aeruginosa Co-Infection Reduces Candida auris Kidney Burden In Vivo

**Figure 7:**
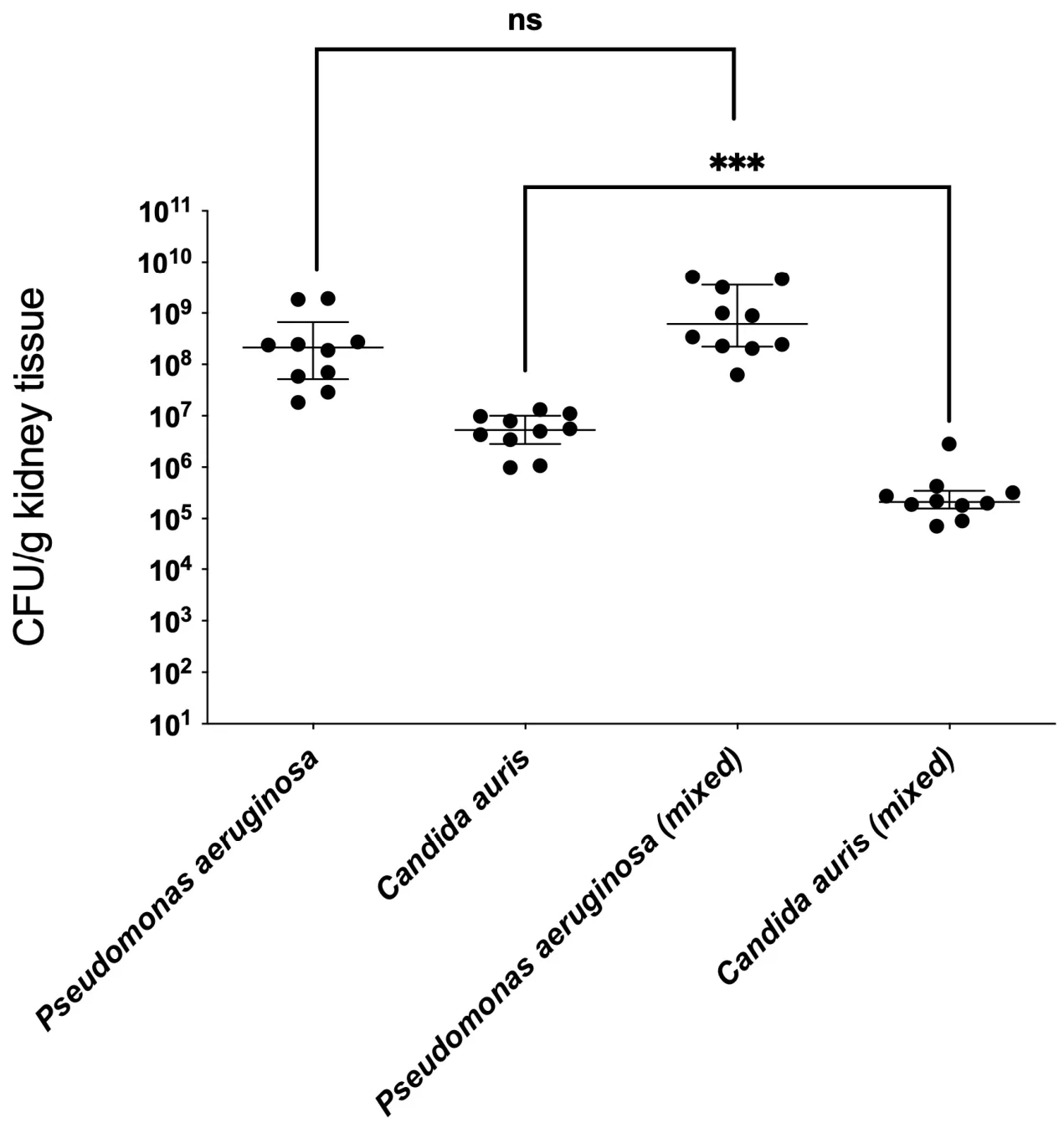
Kidney burden of *C. auris* and *P. aeruginosa* in a systemic mouse infection model. Fungal and bacterial burdens in kidney tissue are expressed as colony-forming units per gram (CFU/g). Mice were infected with *C. auris*, *P. aeruginosa*, or a mixed inoculum containing both organisms. Individual symbols represent individual animals, and horizontal lines indicate the median with interquartile range. Statistical comparisons were performed separately for each microorganism using two-tailed Mann–Whitney U tests. Statistical significance is indicated as *** *p* < 0.001.

To assess whether the antagonistic interaction observed in vitro was associated with altered pathogen burden during polymicrobial infection, fungal and bacterial kidney burdens were quantified in an immunocompromised mouse model (Figure 7). The median *C. auris* kidney burden decreased from 5.3 × 10^6^ CFU/g in monoinfected mice to 2.1 × 10^5^ CFU/g in co-infected mice, corresponding to an approximately 1.4-log_10_ reduction (*p* < 0.001). In contrast, *P. aeruginosa* kidney burdens did not differ significantly between monoinfected and co-infected mice (*p* = 0.089). Therefore, *P. aeruginosa* co-infection was associated with a substantial reduction in *C. auris* kidney burden, while the bacterial kidney burden was not significantly affected by the presence of *C. auris*.

## 4. Discussion

The present study demonstrates that the early interaction with *P. aeruginosa* is associated with less pronounced proliferation of *C. auris* and extensive fungal transcriptional remodelling within 2 h of co-culture. This response involved pathways related to translation, carbon metabolism, stress responses, lipid metabolism, and cell-surface organization. Despite the altered fungal growth phenotype, bulk extracellular glucose concentrations at the 2-h transcriptomic sampling point were comparable between *C. auris* monoculture and co-culture. Thus, the early growth phenotype and associated transcriptional changes occurred in the absence of a detectable co-culture-specific reduction in bulk extracellular glucose concentration at this time point, although alterations in glucose utilization or carbon-metabolic regulation at the cellular level cannot be excluded. This interpretation is consistent with the findings of Macedo et al. (2025), who reported that glucose supplementation did not abolish the antagonistic effect of *P. aeruginosa* against *C. auris* [4]. Similarly, no significant co-culture-associated differences in extracellular iron or zinc concentrations were detected at the 2-h transcriptomic sampling point, although differences became more pronounced at later time points. Finally, the interaction observed in vitro was accompanied by an asymmetric effect in vivo, with *P. aeruginosa* co-infection associated with an approximately 1.4-log_10_ reduction in *C. auris* kidney burden.

Antagonistic interactions between *P. aeruginosa* and *Candida* species are well established and can involve quorum-sensing molecules, secreted metabolites, direct interactions, and nutrient competition [22,25,26,27]. More recently, Macedo et al. (2025) directly demonstrated antagonism between *P. aeruginosa* and *C. auris* and provided evidence for contributions of secreted bacterial products and iron availability [4]. The present study extends these observations by defining the early transcriptional response of *C. auris*, relating it temporally to changes in extracellular nutrient and metal availability, and demonstrating an asymmetric effect on pathogen burden in vivo.

A prominent characteristic of the early interaction was transcriptional remodelling of carbon metabolism. At the transcriptomic sampling point, bulk extracellular glucose concentrations were comparable between *C. auris* monoculture and co-culture. This was accompanied by differential expression of the glycolytic regulators *PFK1*, *PFK2*, and *TYE7*, as well as *PCK1* and *PDC11*. The observed carbon-metabolic transcriptional changes may reflect an adaptive response to bacterial metabolites or other co-culture-associated signals rather than a response to detectable bulk extracellular glucose depletion. Macedo et al. (2025) demonstrated that increased nutrient availability did not eliminate the antagonistic effect of *P. aeruginosa* on *C. auris* and provided evidence supporting the involvement of secreted bacterial products [4]. Furthermore, *P. aeruginosa*-derived phenazines have been shown to alter respiration and carbon metabolism in *C. albicans* [28]. Although phenazine production was not evaluated in the present study, it is plausible that bacterial metabolites similarly contribute to the carbon-metabolic transcriptional response observed in *C. auris* during co-culture.

The coordinated upregulation of *NGT1*, *HXK1*, *DAC1*, and *NAG1* aligns with transcriptional activation of the GlcNAc utilization pathway and may indicate adaptation to alternative carbon sources. GlcNAc also functions as a signalling molecule in *C. albicans*, inducing transcriptional responses independently of its catabolism [29,30,31]. Therefore, the observed expression pattern may result from GlcNAc-mediated regulatory signalling rather than increased GlcNAc utilization. Since GlcNAc availability, uptake, and metabolism were not assessed, these possibilities cannot be distinguished. Additionally, similar extracellular glucose concentrations in monoculture and co-culture conditions suggest that glucose depletion alone does not account for this transcriptional pattern. This observation is consistent with the findings of Macedo et al. (2025), who reported that glucose supplementation did not eliminate the inhibitory effect of *P. aeruginosa* on *C. auris* [4].

Beyond carbon metabolism, the transcriptomic response involved extensive regulation of translation-, stress-, lipid-, and cell-surface-associated processes. Ribosome biogenesis, rRNA processing, and translation were enriched among upregulated genes despite reduced fungal proliferation, indicating substantial reorganization of the translational process. A potential explanation for the observed discrepancy between the upregulation of ribosome- and translation-associated genes and reduced fungal proliferation is that *C. auris* initiates an early adaptive transcriptional response to the altered conditions imposed by bacterial co-culture [32]. The increased transcription of genes involved in ribosome biogenesis and translation may indicate a reorganization of cellular protein synthesis rather than enhanced growth. However, as protein synthesis rates and translational activity were not directly measured, the specific consequences of this transcriptional response have yet to be determined. Stress-associated genes showed heterogeneous regulation, while genes associated with ergosterol and fatty-acid biosynthesis, glucan-mannan biosynthesis, cell-wall organization, and adhesion were predominantly downregulated. Similar regulation of metabolic and lipid-associated pathways has been described during *Candida*–*Pseudomonas* interactions, including in response to *P. aeruginosa*-derived phenazines, which can alter fungal respiration and metabolism [27,33,34,35]. However, these findings should be interpreted as transcriptional pathway responses because phenazine production, respiration, lipid abundance, and cell-wall composition were not directly measured.

RT-qPCR analysis corroborated the RNA-seq-based transcriptional changes, demonstrating a strong positive correlation between the two methods (r = 0.945). The transcription patterns of genes involved in stress responses (*SOD4*, *HSP21*, and *YDJ1*), ergosterol biosynthesis (*ERG24* and *ERG25*), and iron homeostasis (*HMX1*) were consistent across both approaches. However, this validation was limited to 12 selected genes and does not independently confirm the entire transcriptional dataset. While the concordance between RNA-seq and RT-qPCR increases confidence in the observed transcriptional changes, further functional validation is necessary to determine the biological consequences of these changes.

The response to direct bacterial interaction was also distinct from that previously observed following exposure of *C. auris* to exogenous HSL, with limited overlapping differentially expressed genes under the conditions compared [7]. This does not exclude a role for quorum-sensing molecules but indicates that HSL alone does not reproduce the broader transcriptional response elicited by intact *P. aeruginosa*, consistent with the involvement of multiple signals and environmental changes during direct co-culture.

Temporal measurements of metal concentrations indicated that the early transcriptional response occurred before detectable changes in bulk extracellular metal levels. Although *FRE10* and *HMX1* were already downregulated at the transcriptomic sampling point, extracellular iron concentrations did not differ significantly between monoculture and co-culture at this stage. A significant reduction in iron, approximately 49% lower in co-culture, was observed at 4 h. Extracellular zinc was approximately 47% lower in co-culture at 6 h; however, this difference was not statistically significant after correction for multiple comparisons. Iron availability can modulate bacterial–fungal interactions through competition, siderophore-mediated acquisition, and regulation of secreted antifungal factors [9,36,37]. Macedo et al. (2025) demonstrated that iron supplementation partially alleviated the inhibitory effect of *P. aeruginosa* on *C. auris* [4]. The early downregulation of *FRE10* and *HMX1* contrasts with the induction of iron-acquisition-associated responses observed under severe iron limitation in other *Candida* models [36,37], suggesting that this is not a classic response to measurable extracellular iron depletion. It is possible that bacterial interaction alters local iron accessibility or affects fungal iron-associated transcriptional regulation via secreted metabolites. As intracellular iron availability, microbial iron uptake, bacterial siderophores, and individual secreted metabolites were not quantified, these mechanisms remain speculative.

The in vivo findings extend the relevance of the interaction beyond the co-culture system. *P. aeruginosa* co-infection was associated with an approximately 1.4-log_10_ reduction in *C. auris* kidney burden, whereas bacterial burden was not significantly altered. This asymmetric effect is consistent with the broader importance of bacterial–fungal interactions during polymicrobial infection [2]. However, the in vivo experiment does not establish that the reduction in fungal burden is mediated by the same processes observed in vitro; host responses and tissue-specific conditions may also contribute.

Several limitations should be considered. Transcriptomic profiling was restricted to a single early time point and to the fungal response; dual RNA-seq would be required to characterize bacterial transcriptional changes. Extracellular glucose and metal measurements reflect bulk concentrations and do not resolve intracellular or local nutrient availability or distinguish microbial uptake from sequestration. Intracellular metal concentrations and bacterial siderophore production were not measured, and transcriptional changes in lipid and cell-surface pathways were not validated biochemically. Moreover, our study focused exclusively on planktonic cells, and the observed transcriptional responses may differ under dual-species biofilm conditions. These limitations make it difficult to fully interpret the observed early transcriptional response and the reduced fungal burden during co-infection.

## 5. Conclusions

The early interaction between *C. auris* and *P. aeruginosa* is characterized by reduced fungal proliferation and extensive transcriptional remodeling, affecting metabolic, translational, stress-related, lipid, and cell-surface-associated processes. Notably, these early responses occurred without detectable differences in bulk extracellular glucose, iron, or zinc concentrations at the 2-h transcriptomic sampling point. However, changes in extracellular metal profiles became more pronounced as the interaction progressed. The observed reduction in *C. auris* kidney burden during co-infection further supports the biological relevance of this antagonistic interaction in vivo. Collectively, these findings support a multifactorial model of the *C. auris*–*P. aeruginosa* interaction involving transcriptional responses that cannot be explained solely by the measured changes in extracellular glucose, iron, or zinc.

## Data Availability

The RNA-seq data generated in this study have been deposited in the NCBI Gene Expression Omnibus (GEO) database (https://www.ncbi.nlm.nih.gov/geo/; accessed on 8 April 2026) under accession number GSE327064.

## Supplementary Materials

The following supporting information can be downloaded at https://www.mdpi.com/article/10.3390/jof12100758/s1, Figure S1: Propidium iodide (PI) staining of *C. auris* during co-culture with *P. aeruginosa*. Representative images of PI-stained *C. auris* monocultures and *C. auris*–*P. aeruginosa* co-cultures at 0 and 2 h. Heat-inactivated *C. auris* cells served as a positive control. Images are representative of three independent biological experiments. Scale bar = 20 µm. Table S1: Primer pairs used in the study. Table S2: Operating conditions and analytical parameters for inductively coupled plasma optical emission spectrometry (ICP-OES). Table S3: Functional enrichment analysis performed using the Candida Genome Database (http://www.candidagenome.org) and FungiDB (https://fungidb.org/fungidb/app) with default parameters. Terms with adjusted *p*-values < 0.05 were considered significantly enriched.
