# Supplementary material for: Early Transcriptional Adaptation of Candidozyma (Candida) auris During Interaction with Pseudomonas aeruginosa
Source: J Fungi (Basel). 2026 Oct 9;12(10):758. doi: 10.3390/jof12100758 (PMC13706169; doi:10.3390/jof12100758)
Supplement: Supplementary file 1 [file jof-12-00758-s001.zip › TableS1.pdf]

Primer pairs used in the study

Genes	Forward primer (5'-3')	Reverse primer (5'-3')
ACT1 (B9J08_000486)	CTTGTTCCCAGGTATTGC	CTCGTCGTATTCTTGCTTG
TUB2 (B9J08_003245)	GCTGTCCCGTATTGGTAAC	ATTCCTCCCTGATTCTCG
MKC1 (B9J08_002682)	TTACACCGTGACTTGAAACC	CCAAAATACAGCCAACAGAC
YDJ1 (B9J08_005368)	CCACCCAGATAAAAACCC	CATACCGCCCATACCAC
MDR1 (B9J08_003981)	CGACCAAGACCAACCACATTC	CGTATCCGAACACAAAAAGCG
RAS1 (B9J08_003446)	GAACTGCCCCTTTTTAGAGC	ACCATCCACTGCTCCATTAG
UPC2 (B9J08_000270)	GACACCGCCACCTAACCTATC	CCCAAACTGCTCAAATCACC
ERG24 (B9J08_003026)	TGAATCCTGTCACTGAACAC	ACCATCCATATCCTTACCAG
SOD4 (B9J08_001292)	TTACCACGGCTACGAAAAGG	GCACCACCACAGACAAGTTG
ERG25 (B9J08_000367)	CTCTTTCTCGGCTCCTTTC	ATAACGGTGGTGTTGCTTG
HSP21 (B9J08_003627)	GCTTGCTGGAAGAAACACC	GACCGCCTTGTCAAAACC
HMX1 (B9J08_004152)	CGACGAAAACACCTTCAGAC	CGACCACTTCTTCTTGATGC
