# Supplementary material for: Early Transcriptional Adaptation of Candidozyma (Candida) auris During Interaction with Pseudomonas aeruginosa
Source: J Fungi (Basel). 2026 Oct 9;12(10):758. doi: 10.3390/jof12100758 (PMC13706169; doi:10.3390/jof12100758)
Supplement: Supplementary file 1 [file jof-12-00758-s001.zip › TableS2.pdf]

ICP-OES conditions I.

Common conditions	
Replicates	3
Pump speed	15 rpm
Uptake time	25 sec
Rinse time	30 sec
Read time	10 sec
RF power	1.20 kW
Stabilization time	15 sec
Nebulizer flow	0.70 L min^{-1}
Plasma flow	12.0 L min^{-1}
Aux flow	1.0 L min^{-1}
Viewing height	8 mm

ICP-OES conditions II.

Element	Wavelength (nm)	Viewing mode	Stabilization time (sec)
Al	396.152	axial	10
B	249.772	axial	10
Ba	455.403	axial	10
Ca	317.933	axial	10
Cd	226.502	axial	10
Co	228.615	axial	10
Cr	267.716	axial	10
Cu	324.754	axial	10
Fe	238.204	axial	10
K	766.491	axial	10
Li	670.783	axial	10
Mg	280.270	axial	10
Mn	257.610	axial	10
Na	589.592	radial	10
Ni	231.604	axial	10
Pb	220.353	axial	10
Sr	407.771	axial	10
Zn	213.857	axial	10
